# Peritoneal Dialysis with Marked Pneumoperitoneum

**DOI:** 10.1155/2020/1063219

**Published:** 2020-07-20

**Authors:** Norio Nakamura, Masamichi Nakata, Daiki Nagawa, Ikuyo Narita, Takeshi Fujita, Reiichi Murakami, Michiko Shimada, Hirofumi Tomita

**Affiliations:** ^1^Community Medicine, Hirosaki University Graduate School of Medicine, Aomori, Japan; ^2^Department of Cardiology and Nephrology, Hirosaki Graduate School of Medicine, Aomori, Japan

## Abstract

Pneumoperitoneum, the presence of free air within the peritoneal cavity, is often caused by the perforation of gas-containing viscus and commonly requires surgical treatment. However, in patients with peritoneal dialysis, free air is commonly seen on X-ray. We present the case of a patient with peritoneal dialysis with marked pneumoperitoneum. A 75-year-old Japanese male with end-stage renal disease due to antineutrophil cytoplasmic antigen-associated vasculitis had been receiving continuous ambulatory peritoneal dialysis for 9 years. He had a poor appetite and general malaise without abdominal pain or fever. These symptoms gradually worsened, and he was hospitalized. At the time of admission, chest X-ray revealed bilateral free air in the abdomen. Subsequent computed tomography of the abdomen revealed marked pneumoperitoneum. Peritonitis due to perforation of the digestive tract was considered; however, the absence of abdominal pain, fever, and turbidity of dialysis drainage indicated that peritonitis was unlikely. Insufficient air venting during continuous ambulatory peritoneal dialysis bag replacement was suspected. The bag was carefully changed, resulting in a gradual decrease in the free air. We encountered a patient with continuous ambulatory peritoneal dialysis who had significant free air in the abdominal cavity in the absence of peritonitis. The source of the air was determined to be the dialysis bag due to insufficient venting during replacement. This case underscores the importance of instructing patients with continuous ambulatory peritoneal dialysis on the thorough removal of air from the bag during replacement.

## 1. Introduction

Pneumoperitoneum (PP) is the presence of free air within the peritoneal cavity. This condition is often caused by perforation of gas-containing viscus and commonly requires surgical treatment [1]. However, in patients with peritoneal dialysis (PD), free air is commonly seen on X-ray [2]. We present the case of a patient with PD with marked PP.

## 2. Case Presentation

**Figure 1:**
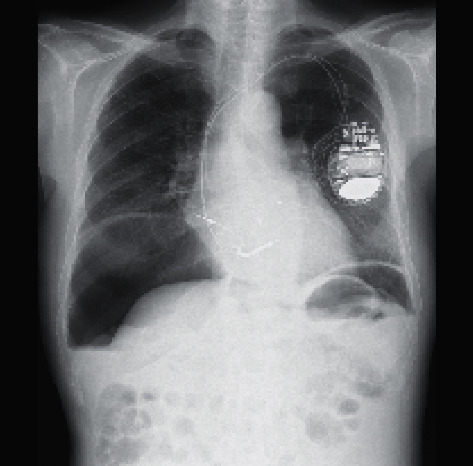
Chest X-ray upon admission. Bilateral free air in the abdomen was observed.

**Figure 2:**
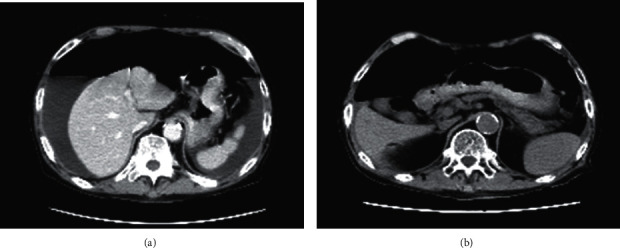
(a), (b) Computed tomography of the abdomen one day after admission. Marked pneumoperitoneum was observed.

A 75-year-old Japanese male with end-stage renal disease due to antineutrophil cytoplasmic antigen (ANCA)-associated vasculitis had been receiving continuous ambulatory peritoneal dialysis (CAPD) for 9 years. He also had an implanted defibrillator (cardio-resynchronized therapy with defibrillator) to treat chronic heart failure resulting from a myocardial infarction. He had a poor appetite and general malaise without abdominal pain or fever. These symptoms gradually worsened, and he was hospitalized. At the time of admission, his blood pressure was 94/52 mmHg, his height was 165 cm, and his weight was 53 kg. At auscultation, no gallop rhythm was heard, and his extremities were not edematous. The laboratory parameters at the time of admission were as follows: white blood cells, 8080/*μ*L; hemoglobin, 13.5 g/dL; platelets, 121 × 10^3^/*μ*L; total protein, 5.2 g/dL; albumin, 1.8 g/dL; lactate dehydrogenase, 309 U/L; glutamate oxaloacetate transaminase, 25 U/L; glutamate pyruvate transaminase, 19 U/L; blood urea nitrogen, 28 mg/dL; creatinine, 4.97 mg/dL; sodium, 125 mmol/L; potassium, 2.7 mmol/L; chlorine, 91 mmol/L; calcium, 6.8 mg/dL; phosphorus, 4.1 mg/dL; C-reactive protein, 0.092 mg/dL; procalcitonin, 0.21 ng/mL; BNP, 319 pg/mL; intact parathyroid hormone, 149 pg/mL; and *β*_2_-microglobulin, 23.4 *μ*g/mL. Chest X-ray revealed bilateral free air in the abdomen (Figure 1). Subsequent computed tomography of the abdomen revealed marked PP (Figures 2(a) and 2(b)).

**Figure 3:**
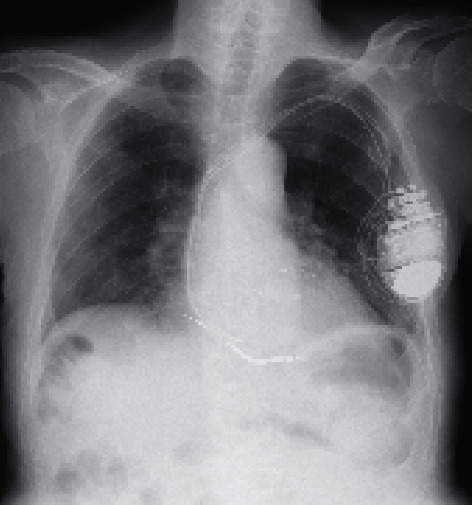
Chest X-ray one day after admission. The free air in the abdomen had disappeared.

Peritonitis due to perforation of the digestive tract was suspected, but the possibility of perforation was considered very low because of the absence of abdominal pain, fever, and turbidity of dialysis drainage. Insufficient priming (air removal) at the time of peritoneal dialysis bag replacement was suspected, and the patient was instructed on how to perform sufficient priming. As a result, the free air in his abdominal cavity gradually disappeared (Figure 3).

## 3. Discussion

We encountered a patient with marked abdominal free air during CAPD. The patient exhibited no pain, fever, or drainage opacification, eliminating peritonitis due to gastrointestinal perforation as the cause. Insufficient air venting during CAPD bag replacement was suspected. The bag was carefully changed, resulting in a gradual decrease in the free air.

In general, free air in the abdominal cavity is a strong indicator of peritonitis due to gastrointestinal perforation [3]. However, this condition has been observed in the absence of gastrointestinal perforation in 20%–23% of patients with PD [2, 4, 5]. Idiopathic PP has been reported, sometimes resulting from iatrogenic causes, including PD [6].

Intraperitoneal free air is an independent risk factor for peritonitis in patients with PD [7] and is thought to be caused by inadequate PD procedures. The most likely cause of intraperitoneal free air in patients with PD is aeration due to insufficient air removal during bag replacement. In the present case, complete air removal during bag replacement led to a gradual decrease in intraperitoneal free air. This case underscores the importance of providing sufficient instructions to patients with CAPD regarding thorough air removal during bag replacement.
